# Can we predict drug response by volumes of the corpus callosum in newly diagnosed focal epilepsy?

**DOI:** 10.1002/brb3.751

**Published:** 2017-06-21

**Authors:** Hyung Chan Kim, Sung Eun Kim, Byung In Lee, Kang Min Park

**Affiliations:** ^1^ Department of Neurology Haeundae Paik Hospital Inje University College of Medicine Busan Korea

**Keywords:** anticonvulsants, corpus callosum, epilepsy

## Abstract

**Objective:**

The aim of this study was to investigate whether volumes of the corpus callosum could predict a response to antiepileptic drugs in patients with newly diagnosed focal epilepsy.

**Methods:**

Fifty‐three patients with newly diagnosed focal epilepsy of unknown etiology and healthy subjects were enrolled in this study. First, we analyzed the differences in the volumes of the corpus callosum between patients with epilepsy and healthy subjects. Second, we divided patients with epilepsy into antiepileptic drug responders and drug nonresponders groups, according to their seizure controls, and evaluated the differences in the volumes of the corpus callosum between the groups. Third, we conducted correlation analyses between the volumes of the corpus callosum and mean diffusion measures in healthy subjects.

**Results:**

The volumes of the corpus callosum in patients with epilepsy were significantly lower than those in normal controls (*p *=* *.0001). Among epilepsy patients, the volumes of the corpus callosum were significantly lower in antiepileptic drug responders compared with nonresponders (*p *=* *.0481), which was the only independent variable for predicting antiepileptic drug response (OR = 10.07, *p *=* *.0434). In addition, we found that the volumes of the corpus callosum were significantly correlated with the mean diffusion measures (fractional anisotropy, *r* = .408, *p *=* *.0027; mean diffusivity, *r* = −0.403, *p *=* *.0028) in normal controls.

**Conclusions:**

We demonstrated that the volumes of the corpus callosum were different according to antiepileptic drug responses in patients with newly diagnosed focal epilepsy, which might suggest that the volumes of the corpus callosum could be a new biomarker for predicting responses to antiepileptic drugs.

## 1 INTRODUCTION

Epilepsy is one of the most common chronic neurological disorders, and the median incidence of epilepsy is approximately 50.4/100,000/year (Ngugi et al., 2011). Antiepileptic drugs (AEDs) are the treatment of choice for most patients with epilepsy, and the number of AEDs is rapidly increasing (McCorry, Chadwick, & Marson, 2004). However, it is known that only approximately 60% of patients with epilepsy respond to the first two AEDs and that less than 4% will respond to further AED trials (Kwan & Brodie, 2000). Recent data have shown that approximately 25% of patients with newly diagnosed epilepsy never become seizure‐free and 16% of patients have a fluctuating course of seizure control (Brodie, Barry, Bamagous, Norrie, & Kwan, 2012).

Early identification of patients who are poorly responsive to AEDs would be essential in patient counseling and selecting patients for more intensive investigations and treatments. Thus, there have been many studies concerning biomarkers for predicting a drug response to AEDs, including disease‐, patient‐ and treatment‐related factors (Kim, Park, Kim, Kwon, & No, 2010; Kwan et al., 2008; Liimatainen, Raitanen, Ylinen, Peltola, & Peltola, 2008; MacDonald et al., 2000; Semah et al., 1998; Siddiqui et al., 2003). Of these factors, the single most important factor for the response to AEDs is the etiology of epilepsy. Many previous studies have consistently demonstrated that the response to AEDs is different depending on the etiology of epilepsy (Liimatainen et al., 2008; Semah et al., 1998). It is well known that focal epilepsy is more difficult to treat than idiopathic generalized epilepsy (Semah et al., 1998). In addition, for focal epilepsy, 40% of patients with an unknown etiology achieved a 12‐month remission over a 2‐year follow‐up period compared with 16% of patients with symptomatic etiologies (Liimatainen et al., 2008). Focal epilepsy of unknown etiology is a common type of focal epilepsy and is presumed to be symptomatic in nature with an unidentified cause. However, the predicting factors for the AED response in focal epilepsy of unknown etiology remain unknown (Kim et al., 2010). Epilepsy is a multifactorial and heterogeneous condition. As such, it is a very useful approach to only study patients with focal epilepsy of unknown etiology because it can make focal epilepsy more homogeneous.

Growing evidence has suggested that epilepsy is a network disease, and focal epilepsy, traditionally considered to be a regional brain disorder, also has widespread alterations of networks beyond the epileptogenic zone (Englot, Konrad, & Morgan, 2016). Many of the studies regarding brain connectivity in epilepsy have demonstrated that focal epilepsy is associated with increases in regional connectivity at the epileptogenic zone, paired with diminished global connectivity (Englot et al., 2016; Kim, Piao, Liu, Bingaman, & Diehl, 2008; Liu et al., 2015; Miro et al., 2015; Weber et al., 2007). However, few studies have investigated the response to AEDs according to brain connectivity, although the number of studies examining brain connectivity in epilepsy has grown rapidly in recent years (Hu et al., 2015; Szaflarski, Kay, Gotman, Privitera, & Holland, 2013).

The corpus callosum is the largest forebrain commissure, with approximately 190 million axons crossing the midline (Unterberger, Bauer, Walser, & Bauer, 2016). Physiological functions are mediated by excitatory and/or inhibitory influences on interhemispheric transfer and include bilateral movements, integration of bilateral sensory and visual information, specialization of language and handedness, emotion, behavior, cognition, memory and complex integrative functions (Unterberger et al., 2016). It has an essential role in the integration of information between the hemispheres as it is the most important connection for global connectivity (Gazzaniga, 2000; Kim et al., 2008). However, no studies have investigated the relationship between the volumes of corpus callosum and global connectivity.

The aim of this study was to investigate whether the volumes of the corpus callosum could predict the response to AEDs in patients with newly diagnosed focal epilepsy of unknown etiology. We hypothesized that the volumes of the corpus callosum could be a new biomarker for predicting AED response in newly diagnosed focal epilepsy of unknown etiology patients. In addition, we evaluated the relationship between the volumes of corpus callosum and global connectivity using diffusion tensor imaging (DTI) in healthy subjects.

## 2 METHODS

### 2.1 Patients

**Figure 1:**
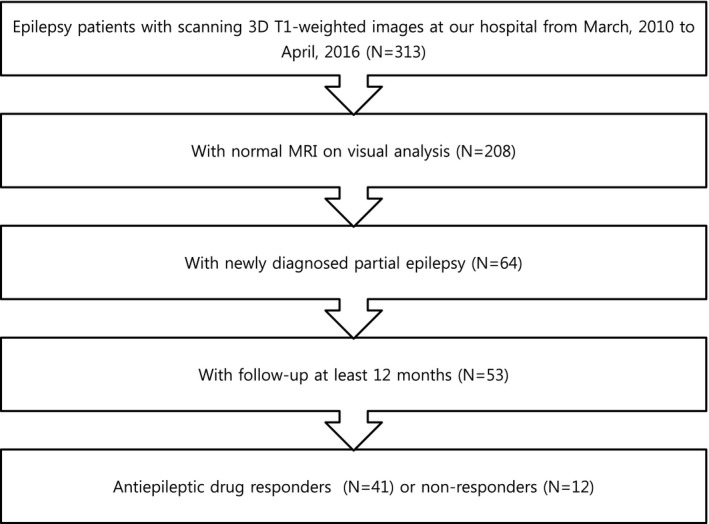
Selection process for patients with epilepsy

This study was conducted with approval from the Institutional Review Board at our institution. This retrospective observational study was performed at a single tertiary hospital. From the epilepsy database of the neurology department, we initially recruited 313 patients with a clinical diagnosis of focal epilepsy based on seizure semiology and EEG findings, who underwent three‐dimensional volumetric T1‐weighted imaging from March 2010 to April 2016. The three‐dimensional volumetric T1‐weighted imaging was suitable for structural volume analysis. Then, we only enrolled patients according to the inclusion criteria as follows: patients (1) without structural lesions on MRI on visual inspections, (2) with newly diagnosed epilepsy, and (3) with a follow‐up of at least 1 year while regularly taking AEDs. Of the 313 patients, 53 patients met the inclusion criteria (Figure 1). Of the 53 patients with epilepsy, 33 patients (62%) were men and 20 patients (38%) were women. The mean age was 35.6 ± 15.8 years. Next, we divided patients with epilepsy into AED responder (*N* = 41) and AED nonresponder (*N* = 12) groups according to their seizure controls. Patients who had successfully achieved at least 6 months of continuous seizure remission were considered to have had a good response to treatment and were consequently placed into the AED responder group.

We collected demographic and clinical data of the patients with epilepsy, including age (age at the time of MRI), sex, age of seizure onset (age at the time of the first seizure), duration prior to diagnosis (duration from the first seizure to starting AED), pretreatment seizure frequency (total seizure frequency before starting AED), pretreatment seizure density (seizure frequency for the previous 6 months before starting AED), EEG findings, and types of AEDs.

### 2.2 Healthy subjects

The control group consisted of 55 age‐ and sex‐matched healthy subjects. Of the healthy subjects, 28 patients (51%) were men and 27 patients (49%) were women. The mean age was 34.3 ± 10.4 years. All subjects had a normal neurological examination and no history of disease, including cardiovascular, neurological or psychiatric disease; diabetes; hypertension; or dyslipidemia. All healthy subjects had a normal MRI on visual inspection.

### 2.3 MRI data acquisition, processing, and analysis to obtain the volumes and mean diffusion measures of the corpus callosum

All the scans were performed using a 3.0T MRI scanner (AchievaTx, Phillips Healthcare, Best, The Netherlands) equipped with an 8‐channel head coil. All the subjects (patients and controls) underwent conventional brain MRI protocols, including axial and coronal two‐dimensional T2‐weighted images, which were obtained using a turbo‐spin echo sequence (repetition time (TR)/echo time (TE) = 3,000/80 ms, slice thickness = 5 mm, echo train length = 14, field of view = 210 mm, matrix size = 512 × 512, number of slices = 24) to exclude subjects with abnormal MRIs on visual inspection. Moreover, all of the patients and healthy subjects underwent sagittal‐oriented high‐resolution contiguous three‐dimensional volumetric T1‐weighted imaging that was suitable for structural volume analysis. The images were obtained using a turbo‐field echo sequence with the following parameters: TI = 1,300 ms, TR/TE = 8.6/3.96 ms, flip angle = 8°, a 1 mm^3^ isotropic voxel size, FOV = 210 × 210 mm^2^, number of slices = 120. In addition, DTI was obtained in healthy subjects. DTI was performed using spin‐echo single shot echo‐planar pulse sequences with a total of 32 different diffusion directions (TR/TE = 8620/85 ms, FA = 90°, slice thickness = 2.25 mm, acquisition matrix = 120 × 120, FOV = 240 × 240 mm2, and *b*‐value = 1,000 s/mm2).

Automated procedures for volumetric measures were carried out as follows. First, image preprocessing was performed, including linear registration, B1 field correction, and nonlinear registration. For linear registration, each volume was rigidly registered with a specific atlas, such as the Talairach space, which was specifically designed to be insensitive to pathology and maximize the accuracy of the final segmentation. Next, any nonhomogenous signal intensity caused by B1 bias field was corrected. High‐dimensional nonlinear morphing to the atlas was then conducted. After image preprocessing, the volume was labeled. To label the structural volume, segmentation was used for three pieces of information to disambiguate the labels: (1) the prior probability of a given tissue class occurring at a specific atlas location, (2) the likelihood of the image intensity given the tissue class, and (3) the probability of the local spatial configuration of labels given the tissue class. We obtained the absolute volumes of the corpus callosum from these automated methods. Next, the volumetric measures were calculated using the following equation to control the differences in the total brain volumes among the subjects: the relative volumes of the corpus callosum (%) = (absolute volumes of the corpus callosum/total intracranial volumes) ×100.

To obtain the mean diffusion measures, including fractional anisotropy (FA) and mean diffusivity (MD), all raw DTI data were preprocessed with FSL (http://www.fmrib.ox.au.uk/fsl). First, eddy current distortions and head motions were corrected by spatially normalizing all of the diffusion‐weighted images. Subsequently, skull‐stripping was applied to exclude nonbrain tissues and regions. Finally, we calculated the mean diffusion measures (FA and MD) in the whole‐brain white matter skeleton for each subject. Volumetric analysis to obtain the volumes of the corpus callosum was performed using the FreeSurfer image analysis suite (version 5.1; http://surfer.nmr.mgh.harvard.edu/).

### 2.4 Statistical analysis

First, we analyzed the differences in the volumes of the corpus callosum between patients with epilepsy and healthy subjects. In addition, we investigated the differences in the volumes of the corpus callosum as well as the demographic and clinical characteristics between drug responders and nonresponders. Comparisons of the demographic and clinical factors were analyzed using the Chi‐squared test for categorical variables and Student's t‐test with a normal distribution or the Mann–Whitney U test without a normal distribution for numerical variables. The differences in the corpus callosum volumes among the groups were analyzed using the Mann–Whitney U test because they were not normally distributed. Multi‐variant analysis was performed using logistic regression analysis. Second, we quantified correlations between clinical variables, including age, age of seizure onset, and pretreatment seizure frequency, and the volumes of the corpus callosum using Spearman's correlation test. Lastly, we conducted a correlation analysis between the volumes of the corpus callosum and mean diffusion measures (FA and MD) in healthy subjects using Spearman's correlation test. Categorical variables are presented as the frequency and percentage. Numerical variables with normal distributions are presented as the mean value ± standard deviation, and those without normal distribution are described as the median value with the range. A *p*‐value less than 0.05 was considered to indicate statistical significance for all calculations. All statistical tests were performed using MedCalc^®^ (MedCalc Software version 13, Ostend, Belgium).

## 3 RESULTS

### 3.1 Epilepsy patients

#### 3.1.1 Differences in characteristics between patients with epilepsy and healthy subjects

**Figure 2:**
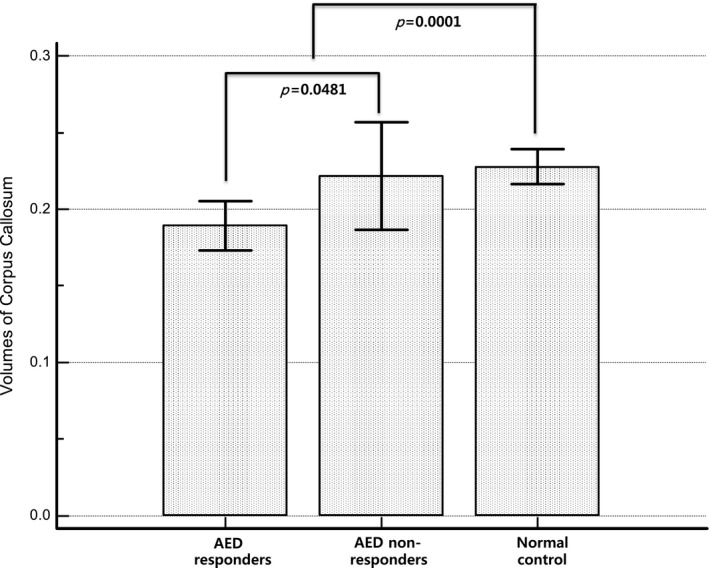
The volumes of corpus callosum. The volumes of the corpus callosum in epilepsy patients are significantly lower than those in normal controls. Among the epilepsy patients, they are significantly lower in antiepileptic drug responders than nonresponders

The age and sex of patients with epilepsy were not different from those of the healthy subjects (Table 1). However, the volumes of the corpus callosum in patients with epilepsy were significantly lower than those in healthy subjects (0.1820 vs. 0.2228%, *p *=* *.0001) (Figure 2). All the patients with epilepsy were on one AED. Thirty‐three patients took voltage‐gated sodium channel blocker‐based AEDs (24 patients with lamotrigine, eight patients with oxcarbazepine, one patient with carbamazepine, and one patient with phenytoin), whereas 19 patients took AEDs with other mechanisms of action (12 patients with levetiracetam, five patients with zonisamide, one patient with valproic acid, and one patient with topiramate). On EEG, 32 patients had normal findings; 13 patients had nonspecific findings, such as focal slowing; and eight patients had epileptiform discharges.

**Table 1:** Differences in the demographic, clinical, and radiologic characteristics between patients with epilepsy and healthy subjects

Variable	Patients with epilepsy (*n* = 53)	Healthy subjects (*n* = 55)	*p*‐value
Age, years (±SD)	35.6 ± 15.8	34.3 ± 10.4	.6052
Male, *n* (%)	33 (62)	28 (51)	.2363
Age of seizure onset, years (±SD)	31.8 ± 15.1		
Pretreatment duration, months (range)	12 (1–360)		
Pretreatment seizure frequency, *n* (range)	4 (1–700)		
Pretreatment seizure density, *n* (range)	2 (1–60)		
Epileptiform discharges, *n* (%)	8 (15.1)		
Volumes of corpus callosum, % (range)	0.1820 (0.1118–0.3551)	0.2228 (0.1481–0.3418)	.0001

#### 3.1.2 Differences in the characteristics between antiepileptic drug responders and nonresponders in patients with epilepsy

There were no significant differences between AED responders and nonresponders according to age, sex, age of seizure onset, duration prior to diagnosis, pretreatment seizure frequency, pretreatment seizure density, or epileptiform discharges on EEG (Table 2). However, the volumes of the corpus callosum were significantly lower in AED responders than nonresponders (*p *=* *.0481) (Table 2) (Figure 2). In addition, multiple logistic regression analysis showed that the volume of the corpus callosum was the only independent variable for predicting the AED response even after correcting for clinical variables (OR = 10.07, *p *=* *.0434) (Table 3). The sensitivity and specificity for distinguishing drug nonresponders from drug responders were 91.7% and 43.9%, respectively, and the positive predictive value and negative predictive value were 32.4% and 94.74%, respectively. There were no significant differences between with or without voltage‐gated sodium channel blocker including lamotrigine, oxcarbazepine, carbamazepine, and phenytoin. (26/41 patients in AED responders vs. 7/12 patients with AED nonresponders, *p *=* *.7517).

**Table 2:** Differences in the demographic, clinical, and radiologic characteristics between antiepileptic drug responders and nonresponders

Variable	AED responders (*n* = 41)	AED nonresponders (*n* = 12)	*p*‐value
Age, years (±SD)	36.3 ± 17.2	33.3 ± 10.2	.5667
Male, *n* (%)	26 (63.4)	7 (58.3)	.2195
Age of seizure onset, years (±SD)	32.1 ± 16.4	30.8 ± 10.2	.7815
Duration prior to diagnosis, months (range)	12 (1–360)	30 (4–96)	.2596
Pretreatment seizure frequency, *n* (range)	4 (1–700)	6 (2–50)	.2004
Pretreatment seizure density, *n* (range)	2 (1–60)	2 (1–10)	.5995
Epileptiform discharges, *n* (%)	7 (17.1)	1 (8.3)	.6651
Volumes of corpus callosum, % (range)	0.1801 (0.1118–0.3353)	0.2188 (0.1478–0.3551)	.0481

**Table 3:** Results of multivariate analysis of variables that are predictive of a poor response to antiepileptic drugs

Independent variable	Adjusted odds ratio	95% confidence interval	*p*‐value
Age of seizure onset	0.980	0.93–1.04	.6426
Duration prior to diagnosis	0.99	0.98–1.00	.4356
Pretreatment seizure frequency	1.00	0.98–1.01	.8464
Volumes of corpus callosum (≤0.1775)	10.07	1.07–94.88	.0434

There were no significant differences between AED responders and normal controls according to age and sex (age, 36.3 ± 17.2 vs. 34.3 ± 10.3 years, respectively, *p *=* *.4776; male gender, 26/41 vs. 28/55, respectively, *p *=* *.2242). In addition, there were no significant differences between AED nonresponders and normal controls according to age and sex (age, 33.3 ± 10.2 vs. 34.3 ± 10.3 years, respectively, *p *=* *.7610; male gender, 7/12 vs. 28/55, respectively, *p *=* *.6434). The volumes of the corpus callosum in AED responders were significantly lower than those in normal controls (0.1801 vs. 0.2228%, *p *<* *.0001), whereas those of the AED nonresponders were not significantly different from those in normal controls (0.2118 vs. 0.2228%, *p *=* *.5452).

#### 3.1.3 Correlation between volumes of corpus callosum and clinical variables in patients with epilepsy

There was no significant correlation between the volumes of the corpus callosum and clinical variables, including age, age of seizure onset, and pretreatment seizure frequency (*r* = .116, *p *=* *.4090; *r* = −.040, *p *=* *.7770; *r* = .099, *p *=* *.4825; respectively).

### 3.2 Healthy subjects

**Figure 3:**
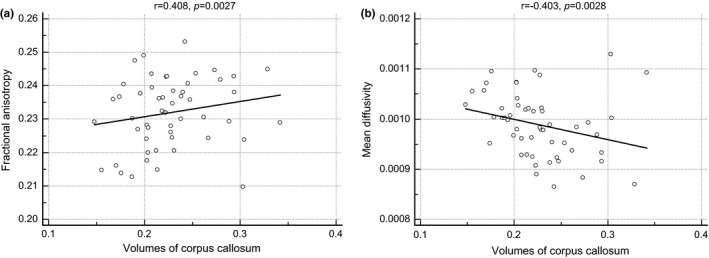
The results of correlation analysis. There are statistically significant correlations between the volumes of the corpus callosum and mean diffusion measures, including (a) fractional anisotropy and (b) the mean diffusivity. AED: antiepileptic drug

We had data for the volumes of the corpus callosum and mean diffusion measures, including FA and MD, in healthy subjects. Thus, we analyzed the correlation between the volumes of the corpus callosum and mean diffusion measures in healthy subjects. We found that the volumes of the corpus callosum were significantly correlated with the mean diffusion measures (FA, *r* = .408, *p *=* *.0027; MD, *r* = −.403, *p *=* *.0028) (Figure 3).

## 4 DISCUSSION

The main finding of this study was that the volumes of the corpus callosum were different according to AED response in patients with newly diagnosed focal epilepsy of unknown etiology. This study was the first to investigate the association between AED response and the volumes of the corpus callosum in patients with focal epilepsy, which might suggest that the volumes of the corpus callosum could be a new biomarker for predicting responses to antiepileptic drugs. In addition, we demonstrated that the volumes of the corpus callous were significantly correlated with the mean diffusion measures, such as FA and MD.

A previous study investigated differences in the gray matter volume according to AED response using voxel‐based morphometry, and it demonstrated that patients with drug‐resistant epilepsy had more widespread gray matter atrophy compared with AED responders (Bilevicius et al., 2010). However, because the previous study enrolled patients with chronic epilepsy, a causal relationship between gray matter atrophy and the AED response was not clear (Bilevicius et al., 2010). It is well known that patients with epilepsy exhibit widespread gray matter atrophy that is negatively correlated with the duration of epilepsy, which may be related to seizure‐induced damage or medication‐related brain atrophy (Bonilha et al., 2006). Furthermore, the seizure frequency is an important factor that can alter functional brain connectivity (Bharath et al., 2015), and AEDs can largely affect the activation of functional networks in patients with epilepsy (Wandschneider et al., 2014). Thus, it was not able to determine if there was a true causal relationship between the AED response and brain connectivity in the study with patients with chronic epilepsy. The strength of our study was that we only enrolled patients with drug‐naïve, newly diagnosed epilepsy.

Epilepsy is reported in up to two‐thirds of patients with developmental corpus callosum agenesis (Taylor & David, 1998). In addition, microstructural changes of the corpus callosum were demonstrated in patients with acquired epilepsy by several studies using DTI and volumetric methods (Kim et al., 2008; Weber et al., 2007). However, although abnormalities of the corpus callosum were frequently reported in patients with epilepsy, it has not been determined whether abnormalities of the corpus callosum were the result or cause of epilepsy. We found that abnormalities of the corpus callosum were found in newly diagnosed epilepsy, which suggests that abnormalities of the corpus callosum might be the cause of epilepsy, not the result of epilepsy.

It remained undetermined why the volumes of corpus callosum were related with AED response. However, one plausible explanation was that the volumes of corpus callosum reflected the global connectivity serving as a potential biomarker for predicting the AED response. It was supported by our present findings that the volumes of the corpus callous were significantly correlated with the mean diffusion measures, such as FA and MD. Although decreased global connectivity in focal epilepsy was already demonstrated by previous studies, it was not clear whether decreased global connectivity represents the damaging consequences of recurrent seizures or an adaptive mechanism to prevent seizure spread out of the epileptogenic zones (Englot et al., 2016). It was a plausible assumption from our study because global connectivity was associated with the adaptive mechanism for preventing seizures; patients with good responses to AEDs had high global connectivity. Although there are few reports of brain connectivity serving as a potential biomarker for predicting the AED response, many studies have revealed that brain connectivity could be a predicting factor for surgical outcome (Keller et al., 2015). Keller et al. (2015) demonstrated that patients with mesial temporal lobe epilepsy with persistent seizures who underwent amygdalohippocampectomy had significant atrophy of the bilateral dorsomedial and pulvinar thalamic regions. They also found that patients with persistent seizures had alterations of DTI‐derived thalamotemporal probabilistic paths bilaterally, relative to those patients who were rendered seizure‐free (Englot et al., 2015; Keller et al., 2015; Xu et al., 2014). Another study demonstrated that patients with unsuccessful surgical outcomes in mesial temporal lobe epilepsy demonstrated larger interhemispheric voxel‐mirrored homotropic connectivity differences than those patients with successful surgical outcomes (Xu et al., 2014). Moreover, a magnetoencephalography study revealed that patients with increased regional connectivity within the resection site were more likely to achieve postoperative seizure freedom than those with neutral or decreased regional connectivity in focal epilepsy (Englot et al., 2015). These previous studies have suggested that brain connectivity can be a potential biomarker for predicting responses to epilepsy treatment. Further researches may be needed to confirm our assumption.

There are several limitations to this study. First, we investigated the differences in demographic and clinical characteristics between AED responders and nonresponders that were considered to be associated with the AED response, such as age of onset, epileptiform discharge, or seizure density. However, no significant differences were detected for these demographic and clinical characteristics between drug responders and nonresponders. This finding may be attributed to a relatively small number of patients with epilepsy. However, this result could be interpreted as indicating that the volumes of the corpus callosum were more sensitive than previous early identification factors. Second, because the follow‐up period of our study was relatively short, the long‐term response to AEDs could not be inferred based only on the present results. Third, we did not have DTI or resting state functional MRI data for patients with epilepsy, only volumetric data. Thus, we did not analyze the relationship between the volumes of corpus callosum and global connectivity in patients with epilepsy. It would be interesting to estimate the global connectivity using DTI or resting state functional MRI in patients with epilepsy, and evaluate the effects of global connectivity to AED response in a further investigation.

In conclusion, we demonstrated that the volumes of the corpus callosum were different according to antiepileptic drug responses in patients with newly diagnosed focal epilepsy, which might suggest that the volumes of the corpus callosum could be a new biomarker for predicting responses to antiepileptic drugs.

## CONFLICT OF INTEREST

None.
